# Physicians in Myanmar Provide Palliative Care Despite Limited Training and Low Confidence in Their Abilities

**DOI:** 10.1089/pmr.2020.0090

**Published:** 2020-12-11

**Authors:** Emily Earl-Royal, Michelle Feltes, Michael A. Gisondi, Loretta Matheson, Maung Ohn “Tony” Htoo, Rebecca Walker

**Affiliations:** ^1^ Department of Emergency Medicine, Department of Emergency Medicine, Stanford University School of Medicine, Palo Alto, California, USA.; ^2^ The Precision Education and Assessment Research Lab, Department of Emergency Medicine, Stanford University School of Medicine, Palo Alto, California, USA.; ^3^ Emergency Department, Parami General Hospital, Yangon, Myanmar.

**Keywords:** barriers, confidence, education, low-income and middle-income countries, Myanmar, training

## Abstract

***Background:*** Patients in low-income and middle-income countries (LMICs) have limited access to palliative care providers. In Myanmar, little is known about physician knowledge of or perceptions about palliative care. An assessment of physician practice and capacity to provide palliative care is needed.

***Objective:*** Our objective was to identify physician practice patterns, knowledge gaps, and confidence in providing palliative and end-of-life care in Myanmar.

***Design:*** This was a cross-sectional survey study.

***Setting/Subjects:*** Participants were physicians practicing in Myanmar who attended the *Myanmar Emergency Medicine Updates Symposium* on November 10 to 11, 2018 in Yangon, Myanmar (*n* = 89).

***Measurements:*** The survey used modified Likert scales to explore four aspects of palliative care practice and training: frequency of patient encounters, confidence in skills, previous training, and perceived importance of formal training.

***Results:*** Study participants were young (median age 27 years old); 89% cared for terminally ill patients monthly, yet 94% reported less than two weeks of training in common palliative care domains. Lack of training significantly correlated with lack of confidence in providing care. Priorities for improving palliative care services in Myanmar include better provider training and medication access.

***Conclusions:*** Despite limited training and low confidence in providing palliative care, physicians in Myanmar are treating patients with palliative needs on a monthly basis. Future palliative care education and advocacy in Myanmar and other LMICs could focus on physician training to improve end-of-life care, increase physician confidence, and reduce barriers to medication access.

## Background

Palliative and end-of-life care are increasingly recognized as basic human rights, consistent with the rights to health care and dignity.^1–5^ However, there is unequal access to palliative care throughout the world, representing a significant health disparity for those with terminal illness. Many patients in low-income and middle-income countries (LMICs) have little or no access to palliative care^5–9^ despite clear and compelling needs. For example, LMICs have an increasing prevalence of chronic noncommunicable disease, childhood cancers, and older population demographics, all of which require additional palliative care services.^10–14^

Myanmar is one such LMIC without broad access to palliative care. Myanmar is located in Southeast Asia and was a British colony from the late 19th century until 1948; subsequently, the country was ruled by a military dictatorship until 2011, when it began instituting political and social reforms.^15^ As described in the Global Atlas of Palliative Care, Myanmar is a country with “isolated palliative care provision…[and has] palliative care activism that is patchy in scope and not well supported.”^5^ The 2015 Quality of Death Index, based on in-country interviews with palliative care experts, found that Myanmar ranked 76 out of 80 countries in overall quality of death, with low scores in multiple domains including community engagement, palliative and health care environment, human resources, and quality of care.^12^ In addition, limited supplies of pain medications and health care funding do not reliably address the needs of their population.^5^

Little is known about Myanmar physicians' training, confidence, perceived importance, and frequency of providing palliative care. However, an accurate understanding of physician knowledge, skills, and attitudes regarding end-of-life care is fundamental to building Myanmar's capacity to meet its palliative care needs.

## Objective

We examined whether physicians in Myanmar routinely provided palliative care and whether they felt adequately trained to do so. We hypothesized that Myanmar physicians would report high frequency of care, low rates of previous training, low confidence in their skills, and high perceived importance of formal training in palliative care.

## Methods

### Design

No prior investigation measured the gap between confidence and training needs in palliative care among Myanmar physicians; thus, there were no prior survey instruments on which to base this study. Accordingly, a survey tool was developed based on a literature review and expert opinion. In consultation with a health sciences librarian, literature review was conducted using Google Scholar™ (Mountain View, CA) with the search terms “data + Myanmar + end of life,” “Myanmar + cancer + palliative,” “palliative + Myanmar,” “end of life + Myanmar,” “needs assessment + palliative + Asia,” and “needs assessment + palliative + provider,” and using all databases in Web of Science (Clarivate Analytics, Philadelphia, PA) for “palliative or end of life + Myanmar + Burma or Burmese.”

Study and survey design included experts who are American physicians with training in palliative medicine and medical education research study design, as well as both Myanmar and American physicians with experience in the practice of medicine and medical education in Myanmar. We created our survey instrument using a modified Delphi approach to optimize content and internal structure evidence. The survey was tested among five authors for item generation, survey functionality, matching of item content to the construct, optimal item phrasing, and overall quality control. The survey was screened and edited by multiple physicians practicing in Myanmar to ensure cultural and clinical relevance. The survey was then piloted within the study team before distribution. Pilot results were checked for consistency, providing some evidence of response process validity. The paper survey was then distributed in person to eligible participants after informed consent was obtained verbally. The survey was open for the two-day study period only. No individual identifying information was recorded.

### Setting/subjects

This was a cross-sectional survey of physicians practicing in Myanmar. Our roster of eligible participants included attendees at *Myanmar Emergency Medicine Updates Symposium* on November 10 to 11, 2018, in Yangon, Myanmar. Participants at this conference represented a broad mix of unspecialized physicians and specialized physicians who were unlikely to have advanced training in areas of palliative care. Participants were limited to physicians in practice; trainees were excluded. This study was reviewed by Stanford University Institutional Review Board and given exempt status, Protocol No. 48211.

### Measurements

Participants were asked to respond to questions about four aspects of palliative medicine using modified Likert scales: frequency of patient encounters, confidence in skills, previous training, and perceived importance of formal training. Participants were also asked to identify the two most important priorities for improving palliative medicine in Myanmar, selected from a list of options; this list included the option “Other” with an open-text response box (Supplementary Appendix SA1, “Palliative Medicine Physician Needs Assessment in Myanmar”).

Data analysis using descriptive statistics was performed in Microsoft Excel 2017 (Redmond, WA) and SAS Enterprise Guide version 8.2 (Cary, NC). Pearson correlations were used to determine significance.

## Results

Eighty-nine participants completed the survey, over half of whom were female (*n* = 48, 54%) (Table 1). Most respondents practiced medicine 10 or fewer years (*n* = 73, 82%) and worked in private hospitals at least part of the time (*n* = 66, 74%). The majority of respondents were unspecialized practitioners (*n* = 59, 66%).

**Table 1.** Participant Demographics

	N (%)
Total	89
Age, (years) median [IQR]	27 [24–33]
Female	48 (54)
Years in practice	
<5	45 (51)
5–10	28 (31)
11–20	11 (12)
21–30	3 (3)
>30	1 (1)
Type of hospital practice	
Public hospital only	25 (25)
Private hospital only	51 (57)
Both public and private	15 (17)
Medical training	
Unspecialized	59 (66)
Specialized	30 (34)
Emergency	13 (15)
Surgery	5 (6)
Family medicine	3 (3)
Internal medicine	3 (3)
Pediatrics	2 (2)
Administration	1 (1)
Anesthesiology	1 (1)
Infectious disease	1 (1)
Orthopedics	1 (1)
Palliative care training	5 (6)

**FIG. 1.**
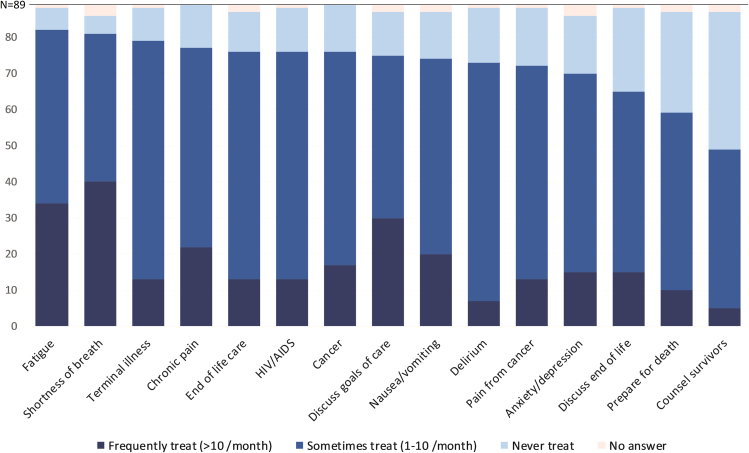
Frequency of treating patients with palliative care.

**FIG. 2.**
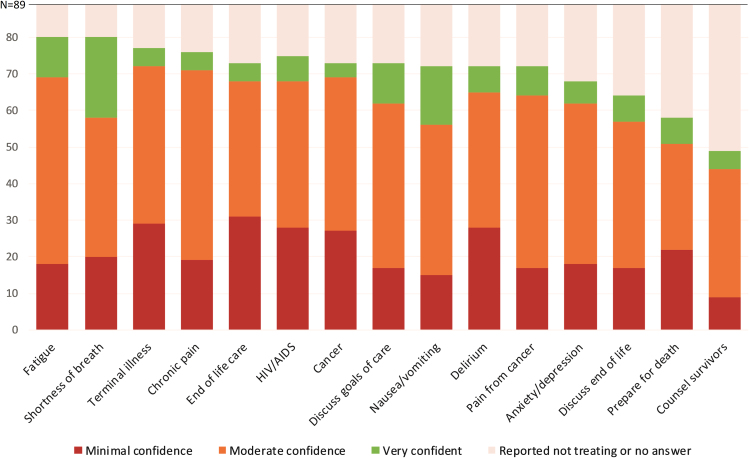
Level of confidence the participants feel in each area.

Most of the study participants treated patients with palliative care needs at least monthly (Fig. 1). The most commonly treated patient symptoms included fatigue (*n* = 82, 92%), shortness of breath (*n* = 81, 91%), and terminal illness (*n* = 79, 89%). Just over half of the study participants provide counseling to survivors (*n* = 49, 55%). Few participants were very confident providing palliative care (Fig. 2). Over one- third of participants reported a lack of confidence providing care for patients with terminal illness (*n* = 29, 34%) or patients at the end of life (*n* = 31, 37%).

**FIG. 3.**
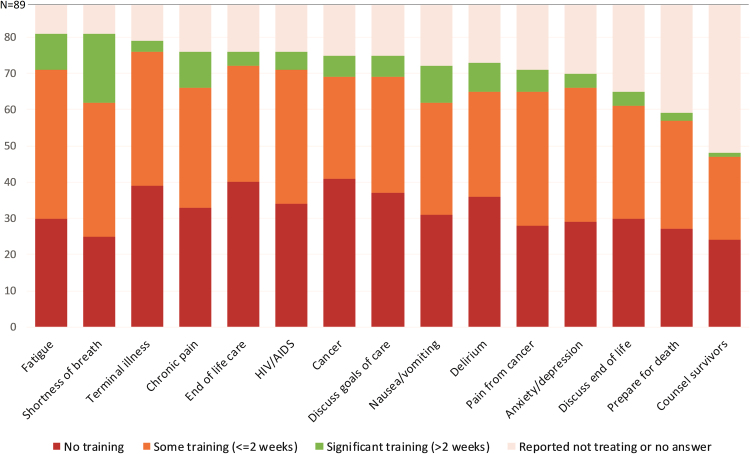
Level of training the participants have received in each area.

**FIG. 4.**
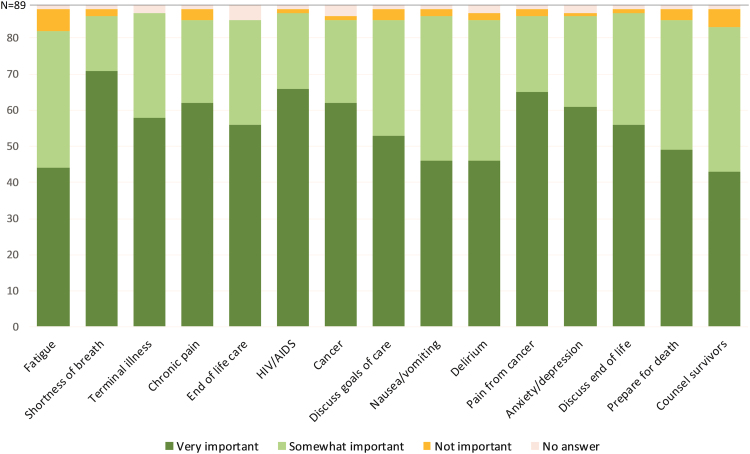
Level of importance the participants assign to receiving training in each area.

Most participants reported zero to two weeks of palliative care training in their careers to date (Fig. 3). Fewer than 1 in 10 participants reported more than two weeks of training in managing terminal illness, helping patients prepare for death, counseling survivors, and end-of-life care. Training and confidence were significantly correlated (*p* < 0.05) in 14 of the 15 domains of palliative care explored in this study. Most participants (*n* = 71, 81%) rated formal training as “important” or “very important” for all 15 domains (Fig. 4).

**FIG. 5.**
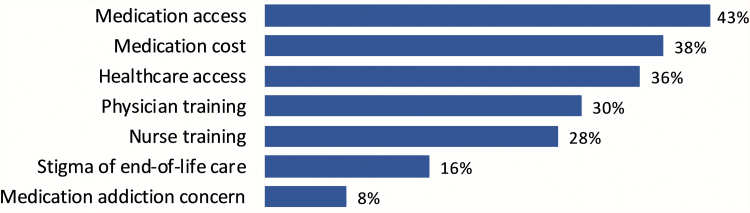
Top priorities for improving palliative care in Myanmar.

The top priorities for improving palliative care services in Myanmar were medication access and cost, health care access, and provider training (Fig. 5).

## Discussion

This study of physician perspectives on palliative care in Myanmar found that most participants provided palliative services at least monthly, most reported little training or confidence in palliative care skills, and nearly all believed that palliative care training is important. It was striking that half of all participants provided end-of-life care without any palliative care training. Top priorities for improving palliative care in Myanmar included barriers to medications and lack of provider training. Our findings highlight the need for improved medical education and health advocacy in palliative care in Myanmar and similar LMICs, namely provider training and medication access and cost.^16^

Although previous studies examined patient-reported needs for palliative care in Southeast Asia and provider knowledge of and attitudes about geriatric palliative care in Vietnam, ours is the first study to report Myanmar physician perspectives on end-of-life and palliative care.^17,18^ Although Myanmar's current palliative care needs are already substantial, the demand for palliative services will only increase over time due to projected changes in demographics.^10–14^ The country's population currently skews very young: 27% of Myanmar's population is <15 years old and only 6% are of age >65 years.^18^ However, Myanmar's population >65 years old is expected to triple after 2050, which will drastically increase its need for elder and palliative care.^19^

We found strikingly low levels of provider confidence and training in key palliative care domains including goals of care, preparations for death, and bereavement counseling for survivors; these gaps extended to conditions of cancer, AIDS, and terminal delirium (Figs. 2 and 3). Within the limitations of our study, confidence in a specific domain is our best measure of a physician's ability to provide satisfactory care. Our data show that Myanmar physicians who reported previous training in certain areas of palliative care were statistically more likely to report confidence in their practice. That is to say, palliative care training correlates with confidence. Our finding is similar to a study of health care providers in Vietnam that found that “good knowledge” about palliative care was associated with a “positive attitude” about it.^18^ Based on our findings, we advocate for better training of Myanmar physicians in the most common domains of palliative and end-of-life care.

Although few physicians in Myanmar are trained in palliative care, local organizations are attempting to address knowledge gaps. The Asia-Pacific Hospice Palliative Care Network and the LIEN Foundation in Singapore are building educational programs in the region, which have trained 28 providers from Myanmar through a partnership with the Myanmar Medical Association.^20^ “Life Asked Death,” a documentary about palliative care needs in Southeast Asia, is raising awareness about end-of-life care in Myanmar.^21^ Critical training in palliative care for Myanmar physicians still depends on foreign aid and the nonprofit sector, including the United States Agency for International Development, Medicins Sans Frontieres, and the U Hla Tung Hospice Foundation.^22,23^

Three themes emerged from our study as priorities for future interventions in Myanmar: provider training, medication access, and medication cost. These themes echo previous studies describing barriers to palliative care in developing countries.^19,22^ Our findings regarding medication access correlate with a longstanding lack of pain medication in LMICs and restrictions on chronic use. Opioid access is unequal around the globe, with 15% of the world's population accounting for 93% of opioid use.^6,10,24–29^ Ironically, Myanmar has a severe shortage of opioid analgesics despite producing much of the world's opium supply.^30^

Our study has several limitations. Our convenience sample may not be representative of physician perspectives throughout Myanmar, and our findings may not generalize to other LMICs. Participants in this study may be younger than average physician age in Myanmar. Although we enrolled participants at an emergency medicine conference, most of the physicians were unspecialized (66%). Similar to other LMICs, many of the “newer” medical specialties, such as emergency medicine and palliative care, are not well established in Myanmar. Our responses may have been affected by sponsorship bias, as some study authors also served as conference faculty. In addition, desirability bias is common among survey respondents. Finally, palliative care is a broad and complex discipline; some important domains related to teamwork, communication, grief, and cultural differences may not have been adequately captured in our survey.

## Conclusion

In summary, Myanmar physicians in our study reported lack of training and minimal confidence across common palliative care domains. Future education and policy interventions should focus on provider training, medication access, and medication cost for Myanmar to better provide palliative and end-of-life care for its citizens.

## Supplementary Material

Supplemental data

## Supplementary Material

Supplementary Appendix SA1

## Abbreviation Used

LMICs: low-income and middle-income countries
