# Supplementary material for: Physicians in Myanmar Provide Palliative Care Despite Limited Training and Low Confidence in Their Abilities
Source: Palliat Med Rep. 2020 Dec 11;1(1):314–20. doi: 10.1089/pmr.2020.0090 (PMC8241381; doi:10.1089/pmr.2020.0090)
Supplement: Supplemental data [file Supp_AppA1.pdf]

Provider Palliative Care Survey

Provider demographics:

1) How old are you?
2) What is your gender? Male / Female / Other ________
3) What is your Provider type? Doctor / Nurse / Prehospital (Ambulance) Provider / Other ___________
4) Are you trained in any medical specialty? Yes/ No If so, which one? ______________
5) How many years have you been in practice? < 5 / 5-10 / 10-20 / 21-30 / >31
6) What is the type of hospital or clinic where you work? Public / Private / Both
7) Have you received any special training on palliative or end of life care? Yes / No If yes, what training ____

**How often do you...**	**Never / Sometimes (1-10 times a month) / Frequently (>10 monthly)**
Care for patients with incurable or terminal illness?	Never / Sometimes / Frequently
Care for patients at the end of life?	Never / Sometimes / Frequently
Care for patients with cancer?	Never / Sometimes / Frequently
Care for patients with HIV or AIDS?	Never / Sometimes / Frequently
Care for patients with chronic pain?	Never / Sometimes / Frequently
Care for patients with pain from cancer?	Never / Sometimes / Frequently
Care for patients with intractable or persistent nausea/vomiting?	Never / Sometimes / Frequently
Care for patients with fatigue?	Never / Sometimes / Frequently
Care for patients with dyspnea/shortness of breath?	Never / Sometimes / Frequently
Care for patients with delirium?	Never / Sometimes / Frequently
Care for patients with anxiety or depression?	Never / Sometimes / Frequently
Have discussions with patients/families about goals of care?	Never / Sometimes / Frequently
Have discussions with patients/families about end of life?	Never / Sometimes / Frequently
Help prepare patients/families for death?	Never / Sometimes / Frequently
Provide care to family members (survivors) after the death of a loved one?	Never / Sometimes / Frequently

**Have you had training to?**	**None / Some(1 day-2 week course) / Significant (>2 week course)**
Care for patients with incurable or terminal illness?	None / Some / Significant
Care for patients at the end of life?	None / Some / Significant
Care for patients with cancer?	None / Some / Significant
Care for patients with HIV or AIDS?	None / Some / Significant
Care for patients with chronic pain?	None / Some / Significant
Care for patients with pain from cancer?	None / Some / Significant
Care for patients with intractable or persistent nausea/vomiting?	None / Some / Significant
Care for patients with fatigue?	None / Some / Significant
Care for patients with dyspnea/shortness of breath?	None / Some / Significant
Care for patients with delirium?	None / Some / Significant
Care for patients with anxiety or depression?	None / Some / Significant
Have discussions with patients/families about goals of care?	None / Some / Significant
Have discussions with patients/families about end of life?	None / Some / Significant
Help prepares patients/families for death?	None / Some / Significant
Provide care to family members (survivors) after the death of a loved one?	None / Some / Significant

Provider Palliative Care Survey

How confident are you to?	Minimal / Moderate / Very
Care for patients with incurable or terminal illness?	Minimal / Moderate / Very
Care for patients at the end of life?	Minimal / Moderate / Very
Care for patients with cancer?	Minimal / Moderate / Very
Care for patients with HIV or AIDS?	Minimal / Moderate / Very
Care for patients with chronic pain?	Minimal / Moderate / Very
Care for patients with pain from cancer?	Minimal / Moderate / Very
Care for patients with intractable or persistent nausea/vomiting?	Minimal / Moderate / Very
Care for patients with fatigue?	Minimal / Moderate / Very
Care for patients with dyspnea/shortness of breath?	Minimal / Moderate / Very
Care for patients with delirium?	Minimal / Moderate / Very
Care for patients with anxiety or depression?	Minimal / Moderate / Very
Have discussions with patients/families about goals of care?	Minimal / Moderate / Very
Have discussions with patients/families about end of life?	Minimal / Moderate / Very
Help prepares patients/families for death?	Minimal / Moderate / Very
Provide care to family members (survivors) after the death of a loved one?	Minimal / Moderate / Very

How important do you think it is to receive formal training in the following areas?	Not at all / Somewhat / Very
Care for patients with incurable or terminal illness?	Not at all / Somewhat / Very
Care for patients at the end of life?	Not at all / Somewhat / Very
Care for patients with cancer?	Not at all / Somewhat / Very
Care for patients with HIV or AIDS?	Not at all / Somewhat / Very
Care for patients with chronic pain?	Not at all / Somewhat / Very
Care for patients with pain from cancer?	Not at all / Somewhat / Very
Care for patients with intractable or persistent nausea/vomiting?	Not at all / Somewhat / Very
Care for patients with fatigue?	Not at all / Somewhat / Very
Care for patients with dyspnea/shortness of breath?	Not at all / Somewhat / Very
Care for patients with delirium?	Not at all / Somewhat / Very
Care for patients with anxiety or depression?	Not at all / Somewhat / Very
Have discussions with patients/families about goals of care?	Not at all / Somewhat / Very
Have discussions with patients/families about end of life?	Not at all / Somewhat / Very
Help prepares patients/families for death?	Not at all / Somewhat / Very
Provide care to family members (survivors) after the death of a loved one?	Not at all / Somewhat / Very

MISC:

8) How would you rate the overall quality of palliative or end of life care in Myanmar?

Excellent / Good / Fair / Needs Improvement / Needs Substantial Improvement

9) What do you think are the <u>top 2</u> most important issues for improving palliative (end of life) care in Myanmar today?

i) Training for Physicians
ii) Training of Nurses
iii) Limited access to see a healthcare provider
iv) Limited availability of medication
v) Cost of medication
vi) Concerns about medication addiction/abuse
vii) Stigma around end of life care
viii) Other ______________________________
